# Supplementary material for: Biocontrol and Plant-Growth-Promoting Traits of Talaromyces apiculatus and Clonostachys rosea Consortium against Ganoderma Basal Stem Rot Disease of Oil Palm
Source: Microorganisms. 2020 Jul 28;8(8):1138. doi: 10.3390/microorganisms8081138 (PMC7463586; doi:10.3390/microorganisms8081138)
Supplement: Supplementary file 1 [file microorganisms-08-01138-s001.zip › Table S1.docx]

**Table S1.** Soil chemical parameters for Bungor soil series used for nursery experiments.

| **pH (in water) (2:5)** | | 4.21 (0.11)* |
| --- | --- | --- |
| **Organic carbon (%)** | | 0.79 (0.06) |
| **Nitrogen (%)** | | 0.09 (0.01) |
| **Phosphorus (mg/kg)** | **Total Phosphorus** | 155.52 (3.32) |
|  | **Available Phosphorus** | 20.11 (1.06) |
| **Exchangeable Cations** | **Potassium** | 0.28 (0.02) |
|  | **Magnesium** | 0.09 (0.01) |
| **Cation Exchangeable Capacity (CEC) (cmol(+)/kg)** | | 8.71 (0.28) |

*Mean of three replicates and numbers in bracket are standard errors.
