# Supplementary material for: Biocontrol and Plant-Growth-Promoting Traits of Talaromyces apiculatus and Clonostachys rosea Consortium against Ganoderma Basal Stem Rot Disease of Oil Palm
Source: Microorganisms. 2020 Jul 28;8(8):1138. doi: 10.3390/microorganisms8081138 (PMC7463586; doi:10.3390/microorganisms8081138)
Supplement: Supplementary file 1 [file microorganisms-08-01138-s001.zip › Supplementary - Figure S2.pdf]

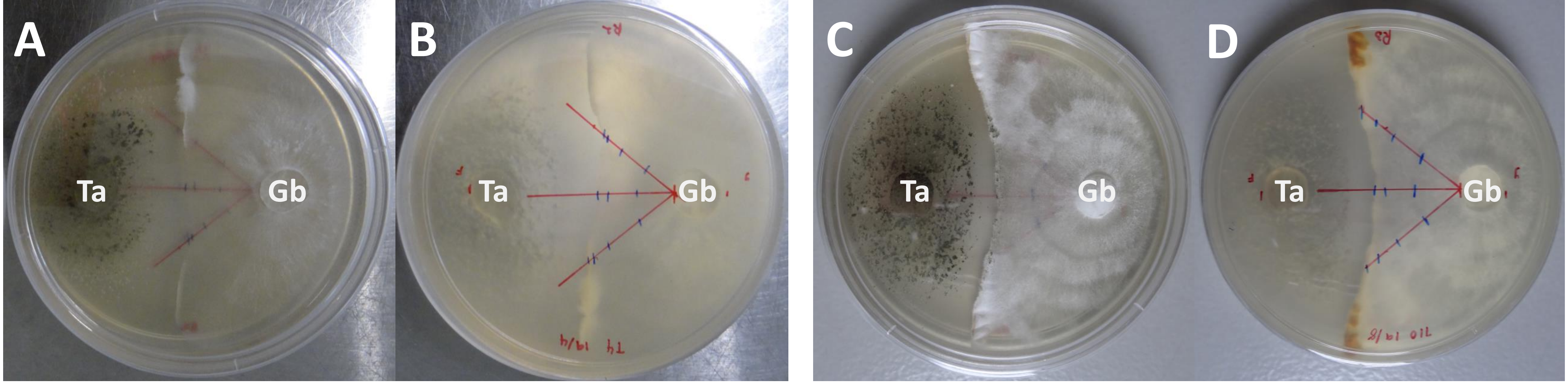

Ta-Gb at 2 WPI (top view) *Ta-Gb* at 2 WPI (bottom view) *Ta-Gb* at 4 WPI (top view) *Ta-Gb* at 4 WPI (bottom view)

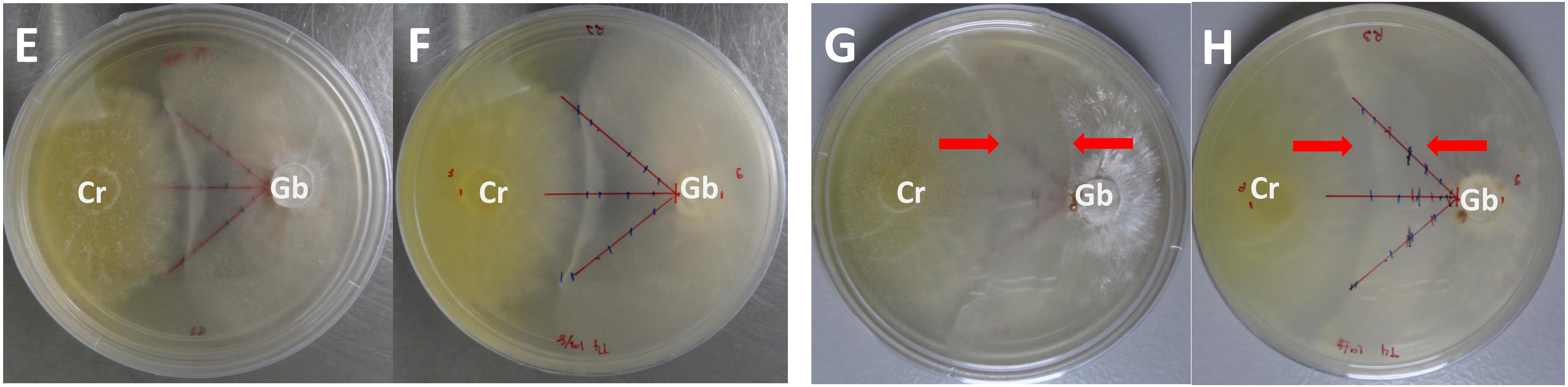

Cr-Gb at 2 WPI (top view) *Cr-Gb* at 2 WPI (bottom view) *Cr-Gb* at 4 WPI (top view) *Cr-Gb* at 4 WPI (bottom view)
