# Supplementary figures and images for: Biocontrol and Plant-Growth-Promoting Traits of Talaromyces apiculatus and Clonostachys rosea Consortium against Ganoderma Basal Stem Rot Disease of Oil Palm
Source: Microorganisms. 2020 Jul 28;8(8):1138. doi: 10.3390/microorganisms8081138 (PMC7463586; doi:10.3390/microorganisms8081138)

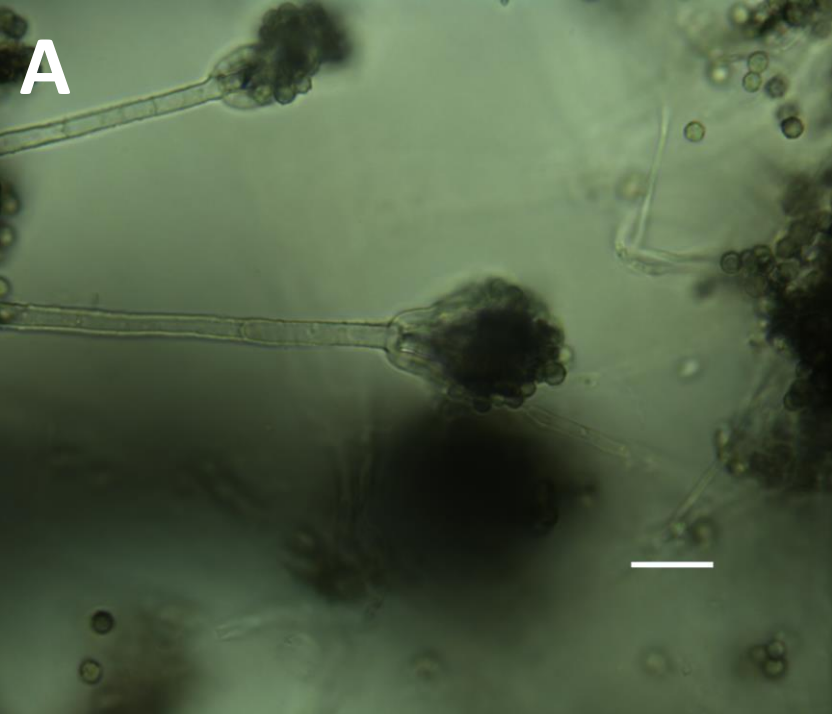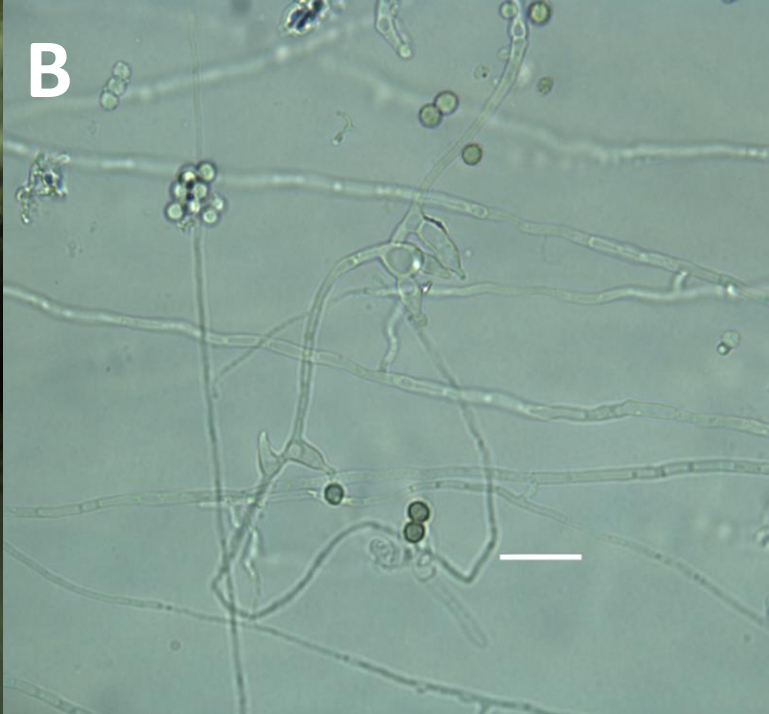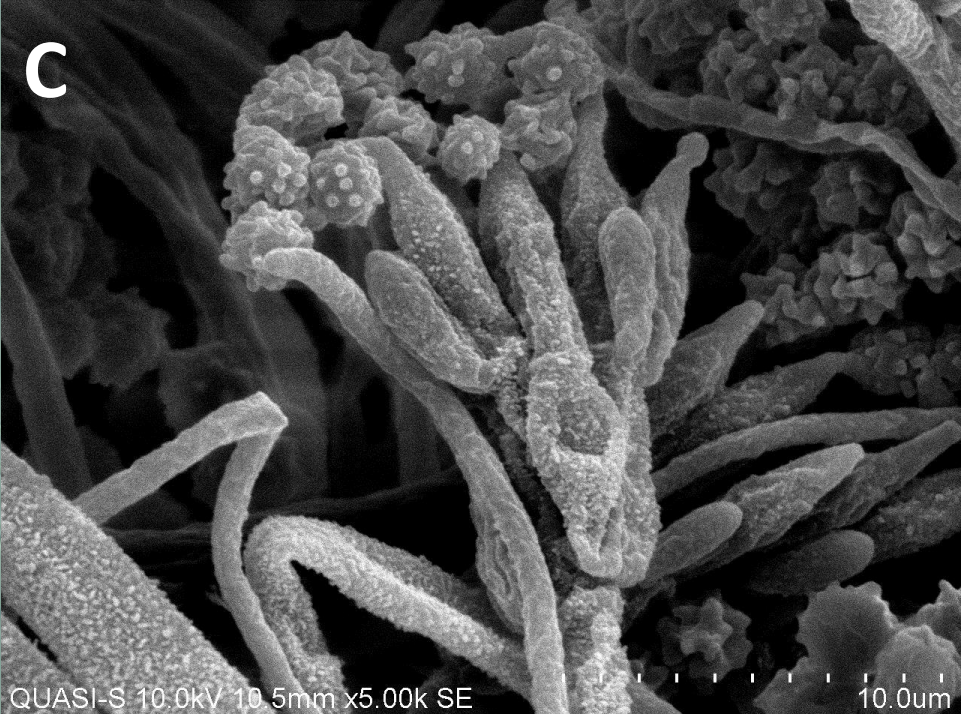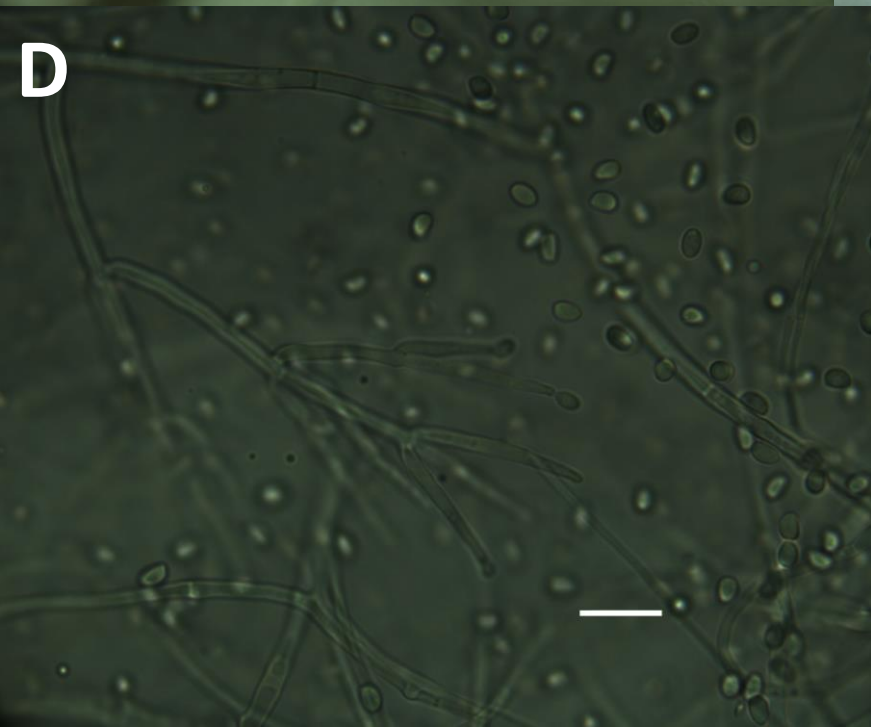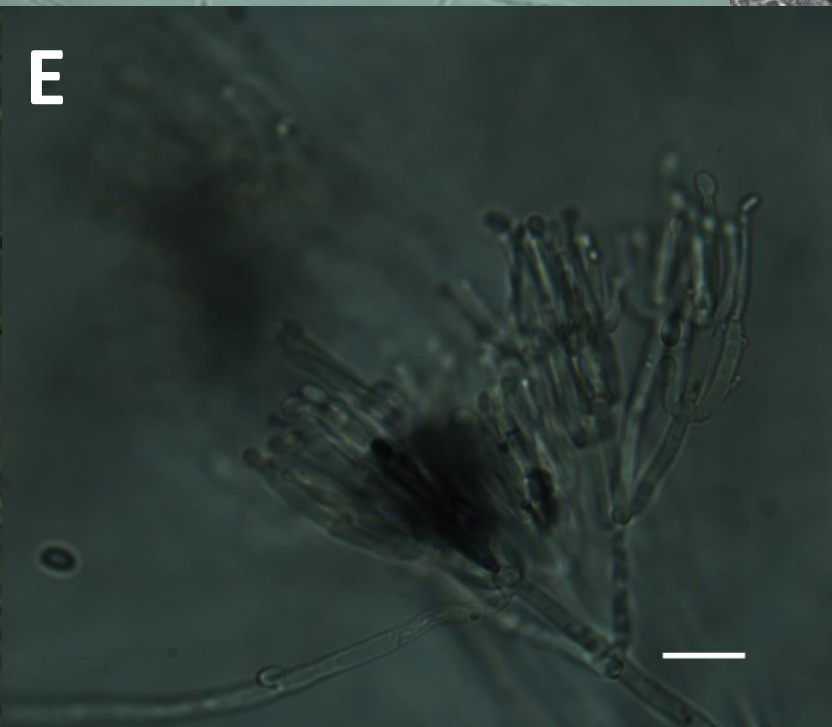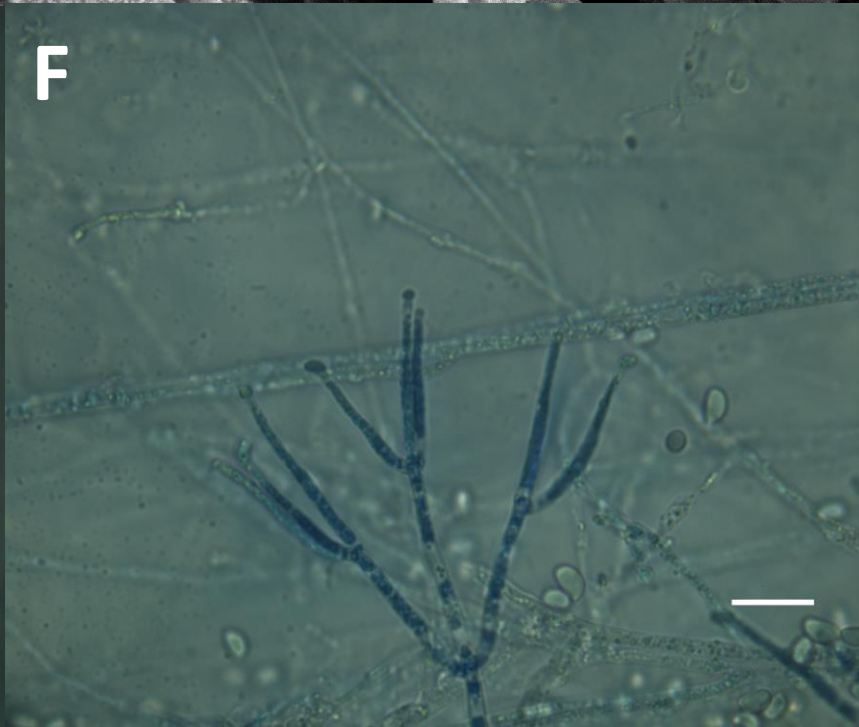

Supplement: Supplementary file 1 [file microorganisms-08-01138-s001.zip › Supplementary - Figure S1.pdf]

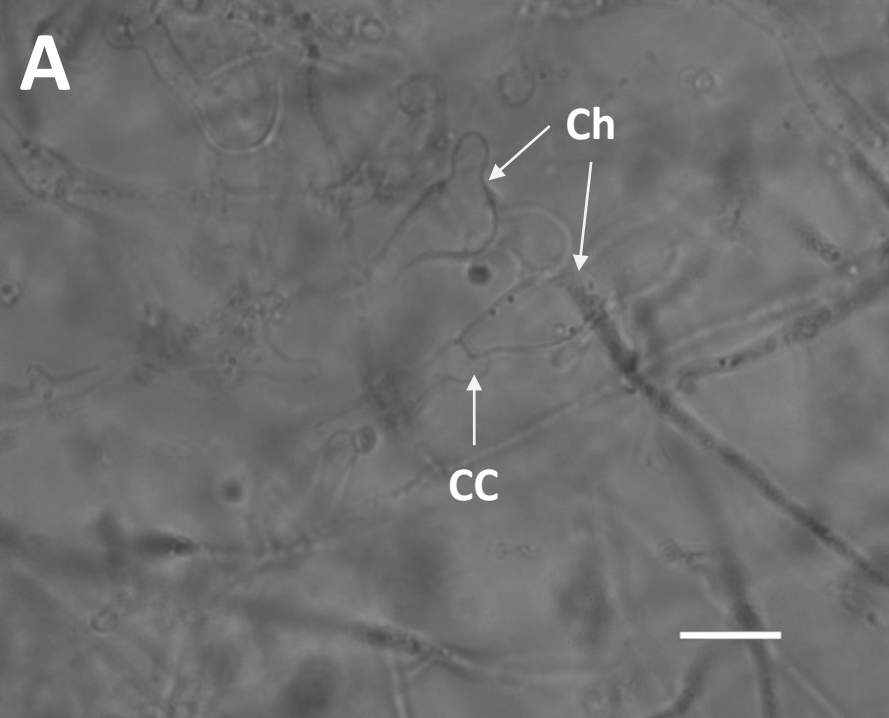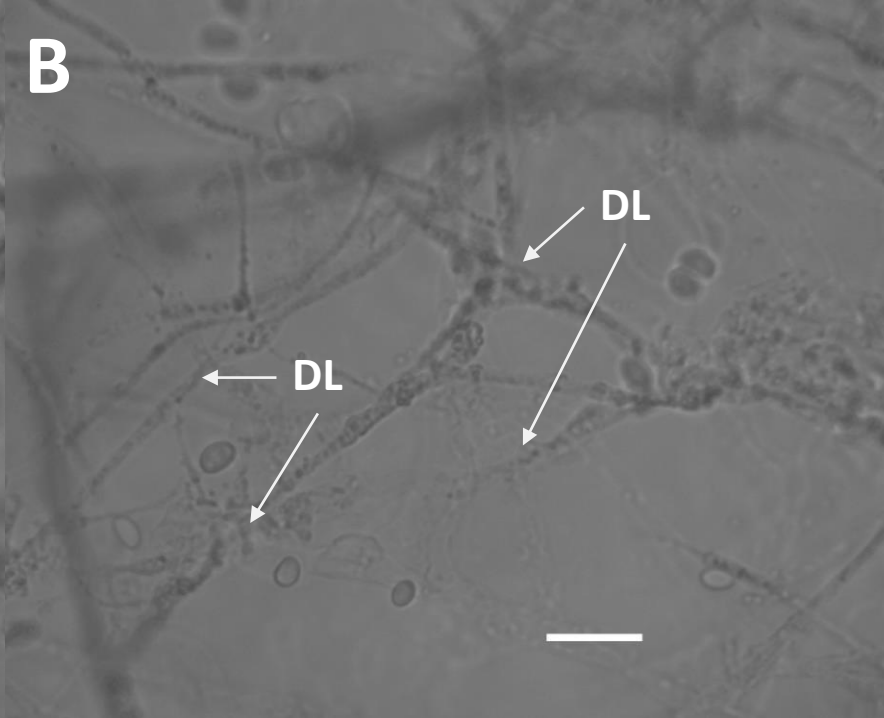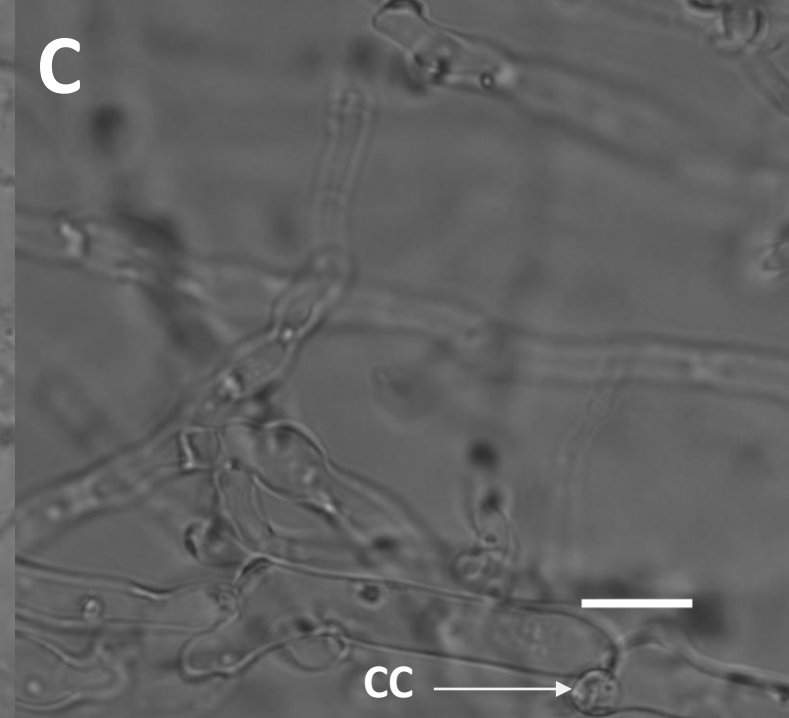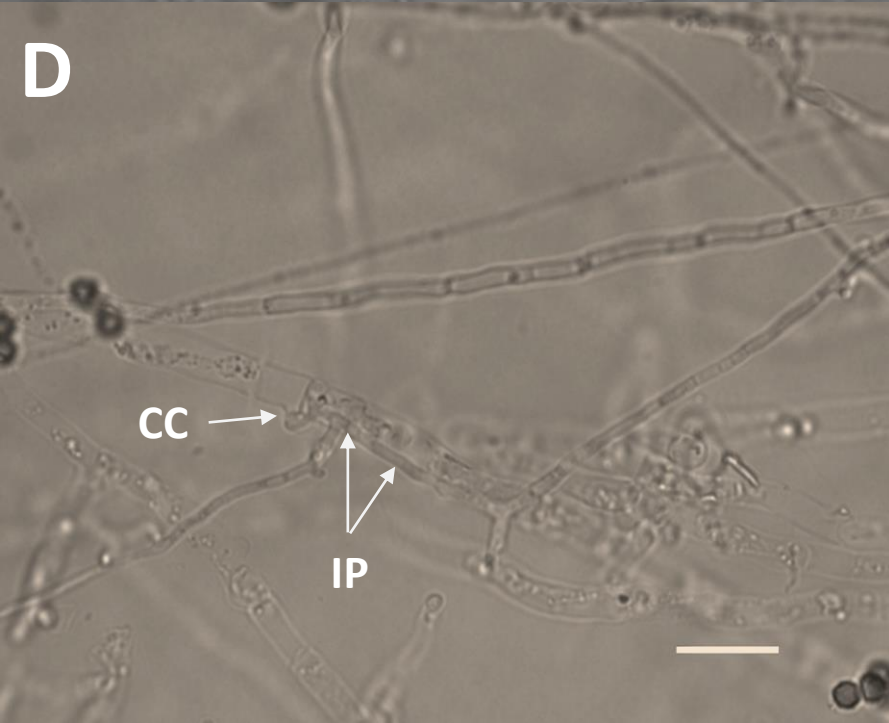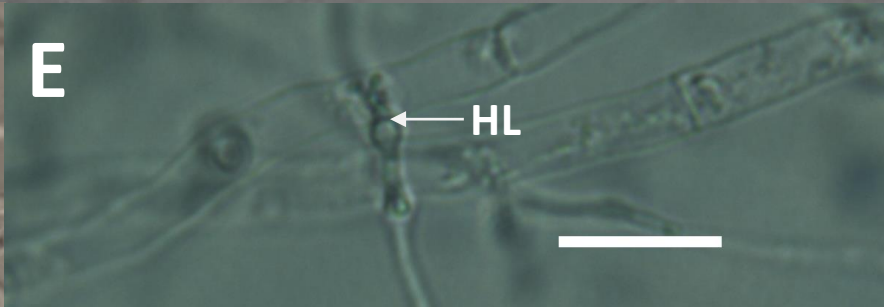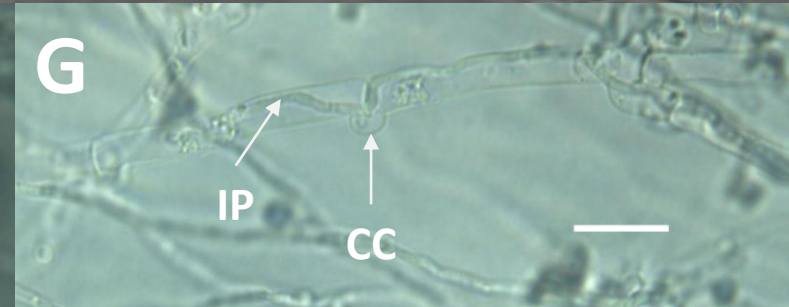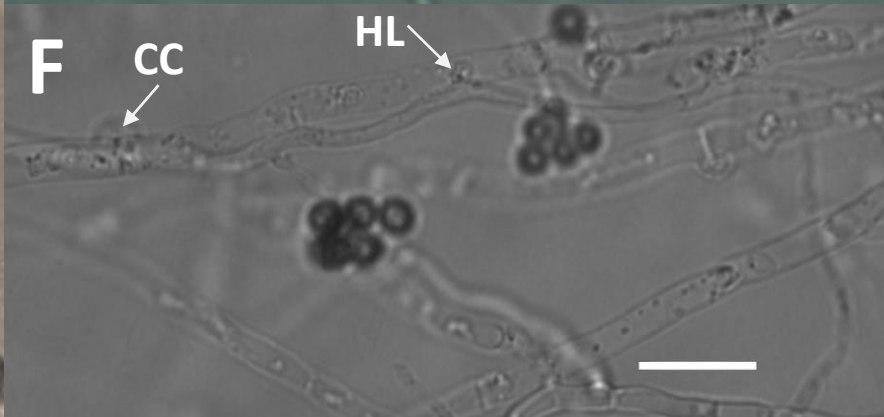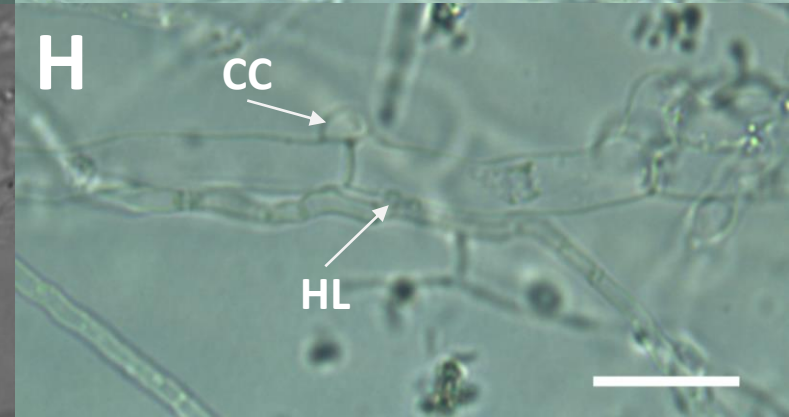

Supplement: Supplementary file 1 [file microorganisms-08-01138-s001.zip › Supplementary - Figure S3.pdf]

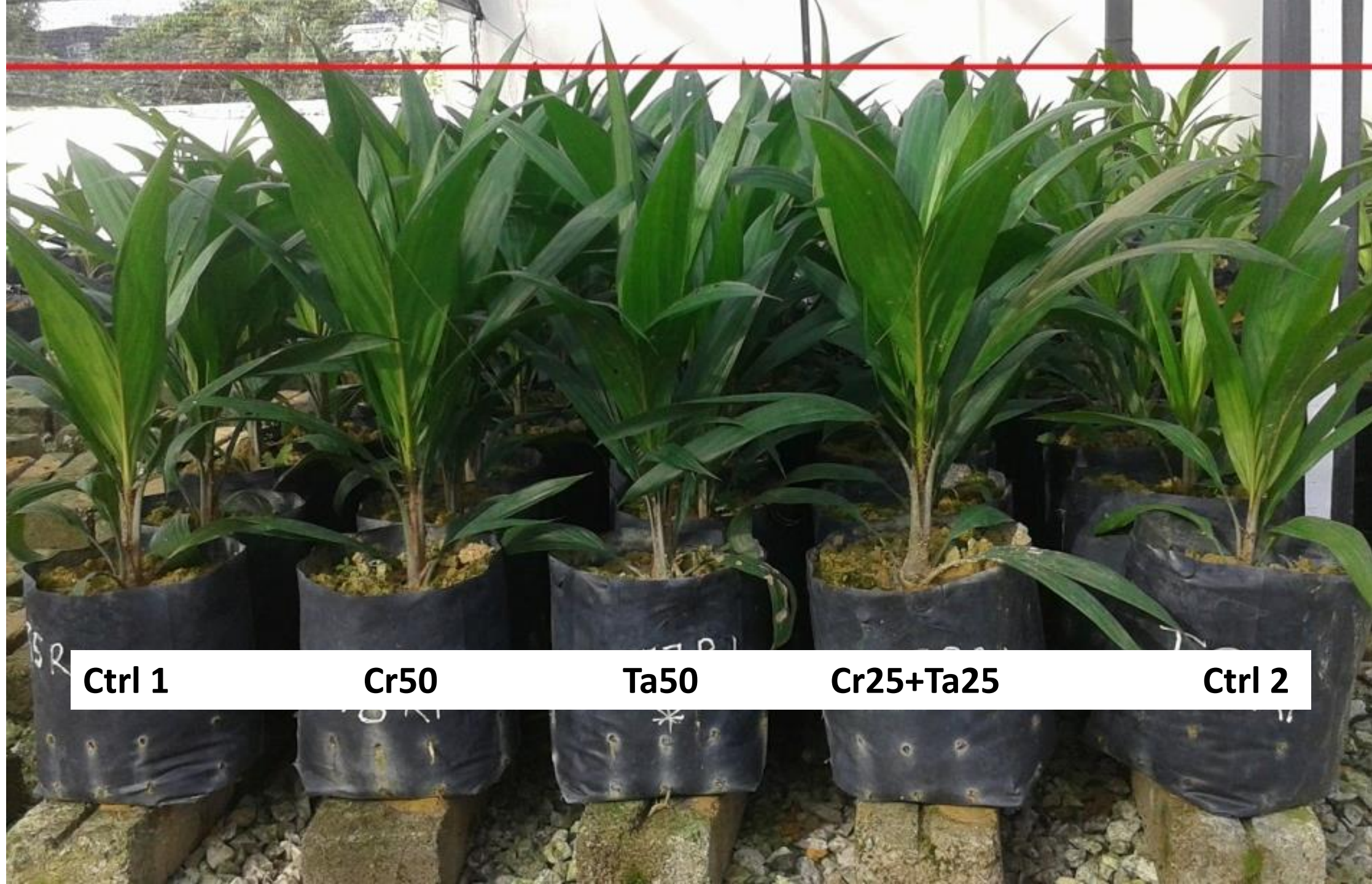

**Ctrl 1**

**Cr50**

**Ta50**

**Cr25+Ta25**

**Ctrl 2**

Supplement: Supplementary file 1 [file microorganisms-08-01138-s001.zip › Supplementary - Figure S4.pdf]
